# Properties of Alkali-Activated Slag Cement Activated by Weakly Alkaline Activator

**DOI:** 10.3390/ma16103871

**Published:** 2023-05-21

**Authors:** Juan He, Shuya Yu, Guochen Sang, Junhong He, Jie Wang, Zheng Chen

**Affiliations:** 1 College of Materials Science and Engineering, Xi’an University of Architecture and Technology, Xi’an 710055, China; m18079691501@163.com (S.Y.); w19309296344@163.com (J.W.); chenz@xauat.edu.cn (Z.C.); 2 School of Civil Engineering and Architecture, Xi’an University of Technology, Xi’an 710048, China; sangguochen@xaut.edu.cn; 3 School of Mathematics and Information Science, Baoji University of Arts and Sciences, Baoji 721013, China; hejunhong@bjwlxy.edu.cn

**Keywords:** Alkali-activated slag (AAS) cement, weakly alkaline activators, setting time, mechanical properties, energy consumption

## Abstract

Sodium sulfate (Na_2_SO_4_) and sodium carbonate (Na_2_CO_3_) are weakly alkaline activators. Alkali-activated slag (AAS) cement prepared with them shows the special advantages of long setting time and low shrinkage, but it shows slow development of mechanical properties. In the paper, Na_2_SO_4_ and Na_2_CO_3_ were used as activators and compounded with reactive magnesium oxide (MgO) and calcium hydroxide (Ca(OH)_2_) to optimize the setting time and mechanical properties. The hydration products and microscopic morphology were also studied using XRD, SEM, and EDS. Furthermore, the production cost and environmental benefits were compared and analyzed. The results show that Ca(OH)_2_ is the main influencing factor for setting time. It reacts preferentially with Na_2_CO_3_ to form CaCO_3_, which makes AAS paste lose plasticity rapidly and shortens the setting time, and then produces strength. Na_2_SO_4_ and Na_2_CO_3_ are the main influencing factors for flexural and compressive strength, respectively. Suitably high content is beneficial to promote the development of mechanical strength. The interaction of Na_2_CO_3_ and Ca(OH)_2_ shows a great effect on the initial setting time. High content of reactive MgO can shorten the setting time and increase the mechanical strength at 28 days. There are more crystal phases in hydration products. Considering the setting time and mechanical properties, the composition of activators are: 7% Na_2_SO_4_, 4% Na_2_CO_3_, 3–5% Ca(OH)_2_, and 2–4% reactive MgO. Compared with ordinary Portland cement (OPC) and AAS cement activated by sodium hydroxide (NaOH, NH) and water glass (WG) with the same alkali equivalent, the production cost and energy consumption are greatly reduced. Compared with P·O 42.5 of OPC, CO_2_ emission is reduced by 78.1%. AAS cement activated by weakly alkaline activators shows excellent environmental and economic benefits and good mechanical properties.

## 1. Introduction

Geopolymers are one of the hot topics in the research of composite materials. Its matrix is composed of a three-dimensional aluminosilicate skeleton. Aluminum and silicon are combined in a tetrahedral skeleton through Si-O and Al-O bonds to form alkali silicaluminate gel [1,2,3]. Alkali-activated slag (AAS) cement is a kind of geopolymer, which is a cementitious material prepared by mixing activators and granulated blast furnace slag (GBFS). Under the action of the activators, the vitreous body of GBFS is destroyed, decomposed, and then polymerized to form hydration products, thus playing the role of cementation [4,5,6,7]. Compared with OPC, AAS cement not only makes use of a large amount of industrial waste slag, but it also presents a simple preparation process without calcination. So, there is no CO_2_ produced by the limestone decomposition, which greatly reduces CO_2_ emissions. Therefore, AAS cement is considered a low-carbon and environmentally friendly cementitious material [8,9].

Activators will affect the hydration process and properties of AAS cement. NaOH (NH) and WG are usually considered to be strong activators. AAS cement prepared with them shows excellent mechanical properties, which has been investigated by numerous scholars. Shi et al. investigated the mechanical properties of AAS mortar activated by NH, and the 28-day compressive strength exceeded 40 MPa when the alkali equivalent was 4–8% [10]. Fang et al. studied the mechanical properties of WG-activated AAS mortar. When the alkali equivalent was 6% and the modulus was 1.2, the compressive strength reached 94.5 MPa at 28 days [11]. However, these strong activators activated AAS cement that sets and hardens quickly, which is not convenient for engineering applications. There is also the problem of large shrinkage. Those problems pose a threat to the long-term property and durability of AAS concrete [12,13,14]. When the alkali equivalent is 4%, the initial setting time of the AAS paste prepared by WG with modulus 1.0 and NH was 15 min and 21 min, respectively [15]. The drying shrinkage of the AAS mortar activated by WG and NH was three times and six times that of the OPC mortar, respectively [15]. In addition, NH is mainly produced by the electrolysis of a saturated NaCl solution, while WG is mainly produced by the hydrothermal method or high-temperature calcination, both of which require a large amount of energy consumption and have low industrial added value. Due to the strong alkalinity of NH and WG, they are highly corrosive and do not have good operability and safety properties in engineering applications [16,17].

Some researchers have added citrogypsum to GBFS-based AAS cement activated with NH or Na_2_SiO_3_ and found that it has a negative effect on the performance [18]. However, it was found that the addition of alkaline activators and citrogypsum to the binding system separately in both cases (AAS cement activated with NH or Na_2_SiO_3_) helps to reduce the initial and final setting times [19]. This provides a new way to adjust the setting time of AAS cement. Furthermore, the possibility of recycling calcined phosphogypsum as a partial replacement for fly ash (FA) in alkali-activated FA (AAFA) paste was studied. The results show that high content of calcined phosphogypsum has a negative effect on the performance of alkali-activated FA [20].

Sodium sulfate (Na_2_SO_4_) and sodium carbonate (Na_2_CO_3_) are weakly alkaline activators compared to WG and NH. Na_2_SO_4_ is a neutral salt and shows a weak excitation effect on AAS. Na_2_CO_3_ is a weakly alkaline salt in its aqueous solution. Rashad found that the mechanical strength of AAS mortar activated by Na_2_SO_4_ is lower than that of OPC [21]. AAS cement prepared with Na_2_CO_3_ shows slow strength development in the early stages and significant strength improvement in the late stages [22,23]. Li and Sun et al. prepared AAS mortar activated by Na_2_CO_3_. The AAS mortar was still in a plastic state at 3 days, while the strength reached 60 MPa at 28 days [23]. The pore solution of AAS mortar activated by Na_2_CO_3_ is less alkaline, so the early hydration is low, while the late strength development is attributed to the formation of carbonate compounds [24]. Although AAS cement activated by Na_2_SO_4_ or Na_2_CO_3_ shows a slow strength development in the early stages, its slow hydration process is conducive to keeping AAS concrete in a plastic state for a long time, facilitating the workability adjustment and construction of an AAS concrete mixture. Moreover, its small shrinkage also contributes to the durability of AAS concrete [15,17].

To enhance the early hydration and mechanical properties of AAS cement activated by Na_2_SO_4_ and Na_2_CO_3_, many scholars have carried out research. The authors’ preliminary tests showed that the AAS paste activated by Na_2_SO_4_ or Na_2_CO_3_ alone did not set and harden and remained in a plastic state after 24 h [25]. When the amount of reactive MgO was 4–6%, the initial setting time of the Na_2_SO_4_ or Na_2_CO_3_-activated AAS paste decreased greatly and the 28-day compressive strength of the Na_2_SO_4_ and Na_2_CO_3_-activated AAS mortar was more than 30 MPa and 45 MPa, respectively [25]. After the incorporation of reactive MgO, the pH of the Na_2_SO_4_ and Na_2_CO_3_-activated AAS suspension can exceed 12. Reactive MgO elevates the pH of the paste and can significantly promote the hydration process of AAS cement activated with Na_2_SO_4_ and Na_2_CO_3_. The formation of hydration products, C-(A)-S-H gel, hydrotalcite, and ettringite (AFt), enhances the compactness of the AAS structure, thus improving the mechanical properties [25]. Abdalqader et al. studied the effect of reactive MgO on a Na_2_CO_3_-activated fly ash–slag composite system. The authors concluded that reactive MgO had a positive effect on the strength development of this cementitious material system and attributed it to the formation of hydrotalcite [26]. Yang et al. blended calcined dolomite (mainly composed of reactive MgO and CaO) into AAS cement activated with Na_2_CO_3_ and found that it accelerated the dissolution of CO_3_^2−^ and promoted the formation of the Mg-Al hydrotalcite phase. When 15% Na_2_CO_3_ was mixed with 10% calcined dolomite, the setting time of the AAS paste was 24 min and the 28-day compressive strength of the AAS mortar was about 40 MPa [27].

The influence of Ca(OH)_2_ on the mechanical properties of AAS cement activated by Na_2_SO_4_ and Na_2_CO_3_ was also studied in the authors’ preliminary experiments. The results showed that the mechanical strength of the AAS mortar activated by Na_2_SO_4_ increased first and then decreased with the increase in Ca(OH)_2_. When Ca(OH)_2_ was 3%, the mechanical properties were the best. The flexural and compressive strength at 28 days were 8.8 MPa and 40.6 MPa, respectively [28], while flash setting occurred when Ca(OH)_2_ was mixed into the Na_2_CO_3_-activated AAS cement [28].The authors concluded that Ca(OH)_2_ reacts with Na_2_CO_3_ to form calcium carbonate, resulting in the abnormally rapid setting of AAS cement. However, when Gao et al. used 8% Na_2_CO_3_ and 2.5% calcium carbide slag (mainly Ca(OH)_2_) as the composite activator, the 1-day and 28-day compressive strengths of the mortar were 21.8 MPa and 37.7 MPa. This indicates that Ca(OH)_2_ and Na_2_CO_3_ can be properly matched to prepare AAS cement to meet the demand [29].

Based on the previous study, the authors used Na_2_SO_4_, Na_2_CO_3_, reactive MgO, and Ca(OH)_2_ as the composite activators and studied the influence of the activators on the setting time and mechanical properties of AAS cement. The main and secondary effects of each activator on setting time and mechanical properties were analyzed by an orthogonal test. A good range for the composition of the composite activators was obtained. Then, the hydration products and microstructure of AAS cements with good mechanical properties were studied using XRD and SEM. Compared with OPC, AAS cement activated by NH and WG, the production cost, CO_2_ emission, and energy consumption were analyzed. It is expected that the research results of the paper will provide a theoretical basis for promoting the engineering applications of AAS cements activated by a weakly alkaline activator.

## 2. Experimentation

### 2.1. Materials

**Figure 1:**
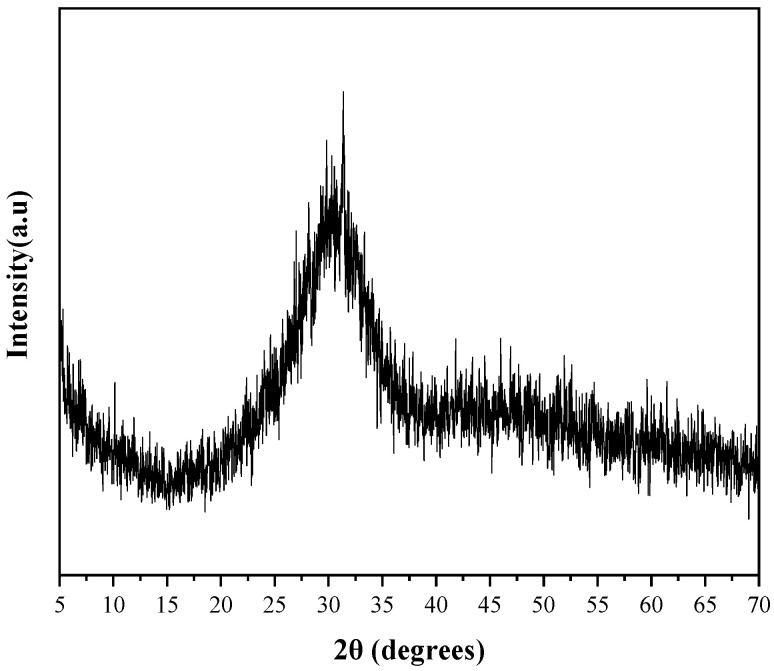
XRD pattern of GBFS.

GBFS was used as the precursor and its main chemical composition is shown in Table 1. Its density is 2.87 g/cm^3^ and the specific surface is 435 m^2^/kg measured by the Blaine method. The basicity coefficient (M = (CaO + MgO)/(SiO_2_ + Al_2_O_3_)) is 1.08. According to GB/T 18046-2017 “Granulated blast furnace slag powder for cement, mortar and concrete” [30], its 7-day and 28-day activity indices were measured to be 85% and 105%. The XRD pattern of GBFS is shown in Figure 1. There is a large dispersion peak at 2θ between 20° and 40°, which indicates that GBFS is mainly composed of a glass phase.

Na_2_CO_3_, Na_2_SO_4_, reactive MgO, and Ca(OH)_2_ were combined as activators. Among them, Na_2_CO_3_, Na_2_SO_4_, and Ca(OH)_2_ are analytically pure reagents in powder form. Reactive MgO is a light-yellow powder and its chemical composition is shown in Table 1. According to YB/T 4019-2006 “Test methods for chemical activity of caustic burned magnesia” [31], its activity value was determined to be 150s. The sand used for the test is natural river sand with maximum particle size and a fineness modulus of 4.75 mm and 2.57. The sand was cleaned and air-dried before use. The water used in the test was tap water.

### 2.2. Experimental Procedures

#### 2.2.1. Experimental Designs

An orthogonal test was used in the experiment, and an orthogonal table of L_25_(5^6^) with six factors and five levels was selected. As shown in Table 2 and Table 3, the factors considered are Na_2_SO_4_, Na_2_CO_3_, reactive MgO, Ca(OH)_2_ and the interaction of Na_2_SO_4_ and Ca(OH)_2_, and the interaction of sNa_2_CO_3_ and Ca(OH)_2_. Each factor has five levels, and the fifth level is the pseudo level. The selected level (activator content) is based on the authors’ preliminary experimental data. The content of the activator (factor) is measured as a percentage of the total amount of GBFS and activator. Table 3 shows that there are 25 combinations of activators. When preparing AAS pastes, the water–binder ratio (W/B) is 0.30. When preparing AAS mortars, the ratio of cementitious material, sand, and water is 1:3:0.5.

#### 2.2.2. Experimental Methods

The AAS paste was prepared according to the Chinese national standard (GB/T 1346-2011) [32]. All powder materials, that is to say GBFS and activator, were first mixed, then mixing water was added, and this was continuously stirred until a homogeneous paste was obtained. The setting time of the AAS paste is determined by Vicat apparatus. The initial setting time is the time from the addition of water to the time when the initial setting needle sinks into the paste 4 ± 1 mm from the bottom of the mold. The final setting time is the time required for the ring attachment to begin to fail to leave a mark on the paste surface. The final results were evaluated using three specimens. 

AAS mortar was prepared according to the Chinese national standard (GB/T 17671-2021) [33]. Sand and all powder materials were first mixed, then mixing water was added and continuously stirred until a homogeneous mortar was obtained. The fresh AAS mortar was poured into a mold of size 40 mm × 40 mm × 160 mm and the mold surface was covered with plastic film. After 1 day of solidification at room temperature, the specimens were demolded and then cured at the condition of 20 ± 2 °C and 95 ± 5RH until the test age. Mechanical properties were carried out at 3 days and 28 days. Strength results were evaluated using three specimens.

X-ray diffraction (XRD) and scanning electron microscopy (SEM) tests were performed with the AAS paste. A paste specimen of 20 mm × 20 mm × 20 mm was prepared and cured at the condition of 20 ± 2 °C and 95 ± 5 RH for 1, 3, 7, and 28 days. Then, the AAS paste specimen was broken into small pieces and soaked in anhydrous ethanol for 3 days to terminate the hydration. Then, the specimen was baked to a constant weight in a vacuum drying oven at 60 °C. The specimen for XRD needs to be ground to pass through a 0.08 mm sieve. The XRD test was performed using a Rikaku D/Max2200 X-ray diffractometer with a Cu-Ka target at a scanning speed of 10°/min. The scanning range was 5° to 70° (2θ) with a resolution of 0.02/step. The dried specimen for SEM needs to be coated with Pt. The SEM test was performed with a ZEISS Gemini 300 SEM equipped with an Oxford X-Max50 energy spectrometer. 

## 3. Results and Discussions

### 3.1. Setting Time of AAS Paste

The results of the setting time and mechanical properties of the 25 groups are summarized in Table 4. It can be seen that the initial setting time of the AAS paste is between 43–81 min and the final setting time is between 53–151 min. N15 shows the shortest initial setting time of 43 min and N7 shows the shortest final setting time of 53 min. Both the initial and final setting times of N19 are the longest, which are 81 min and 151 min, respectively.

The range analysis of the setting time of the AAS paste is shown in Table 5. The correction range value (R′) is calculated according to the following formula:

$$R\prime =d\times R\times \sqrt{r}$$

where: R′—correction range value;

d—correction factor. This is 0.45;

R—Range before correction;

R—The average number of trial replicates per level of a factor. This is 6.

**Table 5:** Correction range value (R′) of setting time for each factor.

Factors	R′ of Initial Setting Time	R′ of Final Setting Time
A (Na_2_SO_4_)	12.01	34.61
B (Na_2_CO_3_)	6.61	27.12
C (Reactive MgO)	11.24	21.38
D(Ca(OH)_2_)	17.86	39.24
A × D (Na_2_SO_4_ × Ca(OH)_2_)	3.22	14.49
B × D (Na_2_CO_3_ × Ca(OH)_2_)	12.16	24.33

It can be seen that the primary and secondary influencing factors of the initial setting time are Ca(OH)_2_ > Na_2_CO_3_ × Ca(OH)_2_ > Na_2_SO_4_ > Reactive MgO > Na_2_CO_3_ > Na_2_SO_4_ × Ca(OH)_2_. The content of Ca(OH)_2_ is the main influencing factor for the initial setting time of the AAS paste, followed by the interaction between Ca(OH)_2_ and Na_2_CO_3_. The interaction between Ca(OH)_2_ and Na_2_SO_4_ has the weakest effect on the initial setting time. The effect of the interaction between Ca(OH)_2_ and Na_2_CO_3_ on the initial setting time is shown in Table 6. It can be seen that the initial setting time is the longest at 80 min with the lowest content of both, and with the highest content of both, the initial setting time is the shortest at 44 min. When the Na_2_CO_3_ content is lowest at 2% at level 1, the initial setting time decreases significantly with the increase in Ca(OH)_2_. When the content of Na_2_CO_3_ is high, the initial setting time does not decrease significantly with the increase in Ca(OH)_2_. When Ca(OH)_2_ content is lowest at 3% at level 1, the initial setting time is shortened significantly with the increase in Na_2_CO_3_. When a high content of Ca(OH)_2_ is added, the initial setting time is shortened with the increase in Na_2_CO_3_. However, when 4% Ca(OH)_2_ at level 2 is added, the initial setting time is extended with the increase in Na_2_CO_3_.

The main order of influencing factors for the final setting time of the AAS paste is Ca(OH)_2_ > Na_2_SO_4_ > Na_2_CO_3_ > Na_2_CO_3_ × Ca(OH)_2_ > Reactive MgO > Na_2_SO_4_ × Ca(OH)_2_. The content of Ca(OH)_2_ is also the main influencing factor for the final setting time, while the effects of the interaction between Ca(OH)_2_ and Na_2_CO_3_ or Na_2_SO_4_ are weaker than their respective effects.

**Figure 2:**
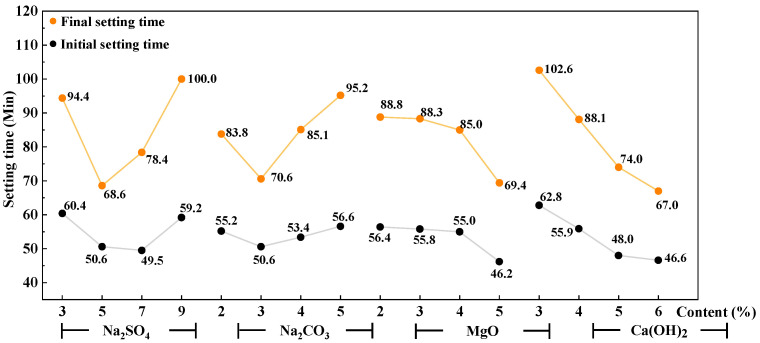
Trend chart of factors influencing setting time of AAS paste.

The trend chart of factors influencing the setting time for the AAS paste is shown in Figure 2. It can be seen that the initial and final setting times decrease first and then increase with the increase in Na_2_SO_4_ and Na_2_CO_3_, and they gradually decrease with the increase in Ca(OH)_2_. The effect of reactive MgO on the setting time is small, and the setting time is significantly shortened only when the content is high, that is, the content is 5% at level 4.

When GBFS, the activators, and water are mixed into the AAS paste, although the solubility of the different activators is different, they will be partially or completely dissolved in the mixing water to form ions, and then chemical reactions will occur. As is shown in Equations (1)–(5), since Ksp (CaSO_4_)/Ksp (CaCO_3_) > 100, Ca^2+^ dissolved by Ca(OH)_2_ will preferentially react with CO_3_^2−^ dissolved by Na_2_CO_3_ to precipitate CaCO_3_, resulting in the loss of plasticity of the AAS paste. The excess Ca^2+^ reacts with SO_4_^2−^ dissolved by Na_2_SO_4_ to form CaSO_4_ precipitate. The molar ratios of Ca(OH)_2_ to Na_2_CO_3_ and Na_2_SO_4_ of 25 combinations of activators are shown in Table 3. Except N2, N3, N4, N6, N7, N9, and N21, n_Ca(OH)2_/n _Na2CO3_ > 1, n_Ca(OH)2_/(n _Na2CO3_ + n _Na2SO4_) < 1. At this point, all the Na_2_CO_3_ participates in the reaction of Ca(OH)_2_ and Na_2_CO_3_, and the excess Ca(OH)_2_ reacts with part of the Na_2_SO_4_ until it is exhausted. The Figure 5 in Section 3.3.1 shows that much calcite is formed at 1 day, along with thenardite, while there are no diffraction peaks of the slaked lime. Therefore, Ca(OH)_2_ is a significant factor affecting the initial and final setting time of the AAS paste, while the interaction between Ca(OH)_2_ and Na_2_CO_3_ shows a stronger effect on setting time than the interaction between Ca(OH)_2_ and Na_2_SO_4_. Moreover, its effect on the initial setting time is more significant. As can be seen from Equations (1)–(5), the reaction between activators simultaneously enhances the alkalinity of the AAS paste system and promotes the destruction, decomposition, and repolymerization of the GBFS vitreous body. So, in summary, with the increase in Na_2_SO_4_, Na_2_CO_3_ content, the setting time decreases. However, with the formation of the more reactive products CaCO_3_ and CaSO_4_, the precipitation will wrap around the slag particles, reducing the hydration rate of the slag. So, when the amount of Na_2_SO_4_ and Na_2_CO_3_ is further increased, the setting time of the AAS paste is prolonged instead.

(1) $${\mathrm{Na}}_{2}{\mathrm{CO}}_{3}\;\overset{\mathrm{Aqueous}}{\to }{\mathrm{Na}}^{+}+{{\mathrm{CO}}_{3}}^{2-}$$

(2) $${\mathrm{Na}}_{2}{\mathrm{SO}}_{4}\overset{\mathrm{Aqueous}}{\to }{\mathrm{Na}}^{+}+{{\mathrm{SO}}_{4}}^{2-}$$

(3) $$\mathrm{Ca}(\mathrm{OH}{)}_{2}\;\overset{Aqueous}{\to }{\mathrm{Ca}}^{2+}+{\mathrm{OH}}^{-}$$

(4) $${\mathrm{Ca}}^{2^{+}}+{{\mathrm{CO}}_{3}}^{2^{-}}\;\overset{Aqueous}{\to }{\mathrm{CaCO}}_{3}↓$$

(5) $${\mathrm{Ca}}^{2^{+}}+{{\mathrm{SO}}_{4}}^{2^{-}}\;\overset{Aqueous}{\to }{\mathrm{CaSO}}_{4}↓$$

Considering the cost, construction, and mechanical properties of AAS cement activated by a weakly alkaline activator, the content of Na_2_SO_4_ and Na_2_CO_3_ should be slightly higher, while the content of reactive MgO and Ca(OH)_2_ should be slightly lower. It is recommended that the content of Na_2_SO_4_, Na_2_CO_3_, reactive MgO, and Ca(OH)_2_ should be 7–9%, 4–5%, 2–4%, and 3–5%, respectively.

### 3.2. Mechanical Properties of AAS Mortar

The results of the mechanical properties of the AAS mortar are shown in Table 4. The 3-day flexural strength is 5–8 MPa, and compressive strength is 18–31 MPa. The 28-day flexural strength is 6–9 MPa, and the compressive strength is 24–45 MPa. The AAS mortar shows good mechanical properties at 3 days. At 28 days of age, the compressive strength of specimens N8, N23, N24, and N25 exceeds 40MPa, and the flexural strength of specimens N23, N24, and N25 is about 9MPa. The range analysis of the mechanical properties is shown in Table 7. It can be seen that the primary and secondary influencing factors of 3-day flexural strength are Na_2_SO_4_ > Na_2_CO_3_ > Ca(OH)_2_ > Na_2_CO_3_ × Ca(OH)_2_ > Reactive MgO > Na_2_SO_4_ × Ca(OH)_2_, and that of 28-day flexural strength are Na_2_SO_4_ > Na_2_CO_3_ > Ca(OH)_2_> Reactive MgO > Na_2_CO_3_ × Ca(OH)_2_ = Na_2_SO_4_ × Ca(OH)_2_. The main factor influencing the flexural strength of the AAS mortar is Na_2_SO_4_, followed by Na_2_CO_3_. The interaction effects of Ca(OH)_2_ with Na_2_CO_3_ or Na_2_SO_4_ are weaker than their respective effects.

The order of influencing factors on the 3-day compressive strength of the AAS mortar is Na_2_CO_3_ > Ca(OH)_2_ > Na_2_CO_3_ × Ca(OH)_2_ > Na_2_SO_4_ > Na_2_SO_4_ × Ca(OH)_2_ > Reactive MgO, and that on 28-day compressive strength is Na_2_CO_3_ > Reactive MgO > Na_2_SO_4_ > Ca(OH)_2_ > Na_2_SO_4_ × Ca(OH)_2_ > Na_2_CO_3_ × Ca(OH)_2_. The main factor affecting compressive strength is Na_2_CO_3_. Ca(OH)_2_ is the next factor affecting the 3-day compressive strength, and the next factor affecting the 28-day compressive strength is reactive MgO. The interaction effect of Ca(OH)_2_ and Na_2_CO_3_ on the compressive strength of the 3-day is higher than that of the 28-day.

**Figure 3:**
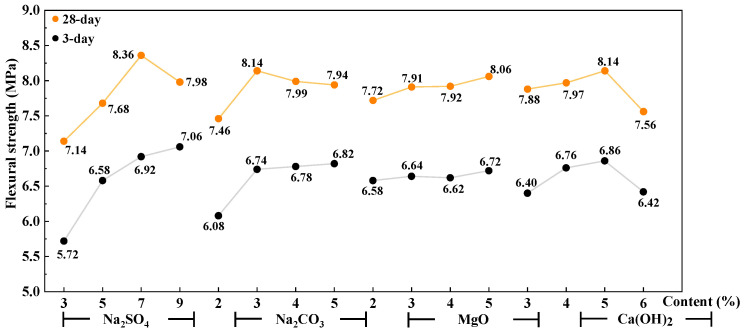
Trend chart of factors influencing flexural strength of AAS mortar.

**Figure 4:**
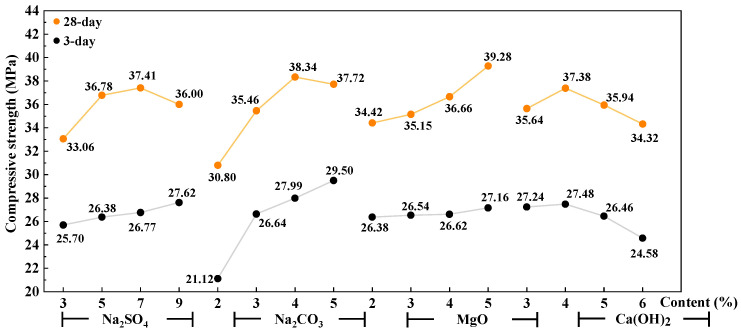
Trend chart of factors influencing compressive strength of AAS mortar.

The trend chart of factors influencing the compressive strength of the AAS mortar is shown in Figure 3 and Figure 4. It can be seen that with the increase in Na_2_SO_4_ and Na_2_CO_3_, the mechanical strength (both flexural and compressive strength) of 3 days of AAS mortar increases, while that of 28 days increases first and then decreases. With the increase in reactive MgO from 2% to 5%, the mechanical properties increase gently except for the obvious increase in compressive strength at 28 days. With the increase in Ca(OH)_2_, the flexural and compressive strengths of the AAS mortar increase first and then decrease.

When the AAS cement comes into contact with the mixing water, calcium carbonate and calcium sulfate are formed due to the reaction between the ions of the activators. The alkalinity of the AAS paste rises, which promotes the destruction and decomposition of the GBFS vitreous, releasing Ca^2+^, Mg^2+^, AlO_4_^5−^, and SiO_4_^4−^, which further reacts to form AFt, gaylussite, C-(A)-S-H gel, and hydrotalcite. So, the AAS cement paste slowly gains mechanical strength. The form of CaCO_3_ contributes a lot to the compressive strength, which has a great influence on the compressive strength both at an early stage and 28 days. Ca(OH)_2_, especially, has a great influence on the 3-day compressive strength.

With the increase in Na_2_SO_4_ and Na_2_CO_3_, the reaction between the ions of the activators is enhanced, and the increase in system alkalinity also promotes the hydration of GBFS, so the mechanical strength increases. As the content of Na_2_SO_4_ and Na_2_CO_3_ continues to increase, products of CaCO_3_, Aft, et al., form a dense protective film on the slag surface, which causes the hydration to be smooth and the hydration degree to decrease, resulting in the reduction of the mechanical strength [34]. With the increase in Ca(OH)_2_ content, the excess Ca(OH)_2_ exists in the form of hexagonal platelet portlandite crystals, which will also cause the reduction of mechanical strength of the AAS mortar [35].

The incorporation of reactive MgO is conducive to the formation of hydrotalcite, which is a substance with a dense microstructure and a slightly increased volume [25]. The formation of hydrotalcite increases the compactness of the matrix, which helps to improve the mechanical properties of the AAS mortar. In general, hydrotalcite is formed when the hydration degree is high. Therefore, the effect of reactive MgO on the setting time is weak, while the improvement of the mechanical properties at 28 days is obvious. The setting time can be greatly reduced only when the content of reactive MgO is high.

According to the range analysis and the variation trend of influencing factors, the content of Na_2_SO_4_ is recommended to be 7% according to the flexural strength. The content of Na_2_CO_3_ is recommended to be 4–5% according to the compressive strength. Considering both the flexural and compressive strength, the content of Ca(OH)_2_ is 3–5% and that of reactive MgO is 2–4%. As can be seen from Section 3.3.1, after reactive MgO is involved in the formation of hydrotalcite, there is still a surplus. So, its content may be lower. The test results of several groups within the content range are summarized as shown in Table 8. It can be seen that the setting time of the five groups of specimens is reasonable, and the mechanical strength is high. Na_2_SO_4_ in N_8_ is 5%, and the reactive MgO content is 5%, but it also shows good properties, which are included in Table 8. High reactive MgO makes N_8_ show high 28-day compressive strength.

### 3.3. The Microstructure of AAS Paste

#### 3.3.1. XRD of AAS Paste

**Figure 5:**
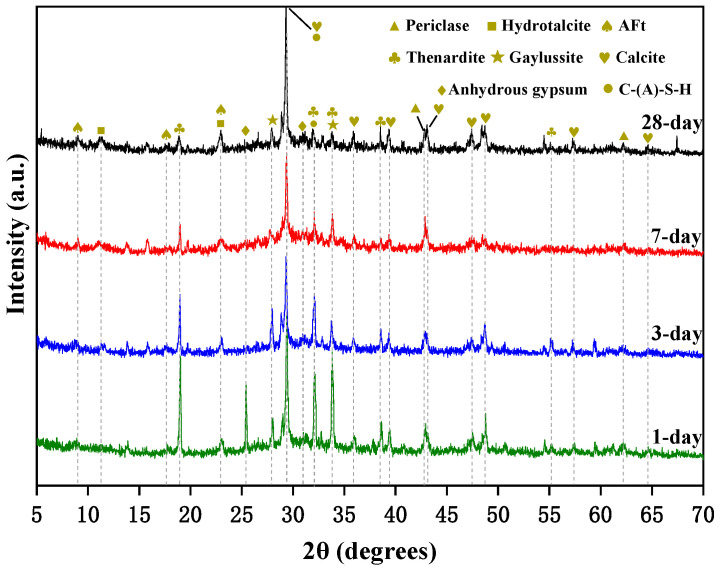
X-ray diffraction patterns of specimen N25.

The XRD pattern of specimen N25 is shown in Figure 5. It can be seen that the main hydration products are C-(A)-S-H gel, calcite (CaCO_3_), Aft (CaO·Al_2_O_3_·3CaSO_4_·31H_2_O), and hydrotalcite (Mg_6_Al_2_(OH)_16_CO_3_·4H_2_O). The unreacted reactive MgO and Na_2_SO_4_ appear as periclase (MgO) and thenardite (Na_2_SO_4_). Compared with the AAS cement activated by WG and NH, there are more crystal phases in the hydration products of AAS cement activated by a weakly alkaline activator. 

At the age of 1 day, a high diffraction peak of calcite appears until the age of 28 days. This is due to the precipitation of CaCO_3_ formed by the reaction of Ca(OH)_2_ with Na_2_CO_3_. The diffraction peak of thenardite and anhydrous gypsum is due to the reaction of part of Na_2_SO_4_ with Ca(OH)_2_ to form anhydrous gypsum, and part of Na_2_SO_4_ still exists in the form of thenardite. With the age from 1 to 28 days, the diffraction peak of thenardite decreases, and that of anhydrous gypsum decreases until it disappears, while that of AFt gradually increases. This is because with the increase in age, AFt is formed by the reaction between sulfate and AlO_4_^5−^ decomposed from GBFS. No diffraction peak of Ca(OH)_2_ is found from the first day to 28 days, indicating that Ca(OH)_2_ is fully involved in the reaction at an early stage. So, it has a significant effect on setting time. The diffraction peak of hydrotalcite is very weak at 1 day, and its peak gradually increases with the increase in age. The diffraction peak of periclase (MgO) decreases with age and is still observed at 28 days. This indicates that the high hydration degree is beneficial to the formation of hydrotalcite, and reactive MgO is surplus, so its content can be reduced. In addition, it is also found that the diffraction peak of gaylussite (Na_2_Ca(CO_3_)_2_·5H_2_O) decreases with the increase in age because the gaylussite may be converted into a more stable calcite [21].

#### 3.3.2. SEM of AAS Paste

**Figure 6:**
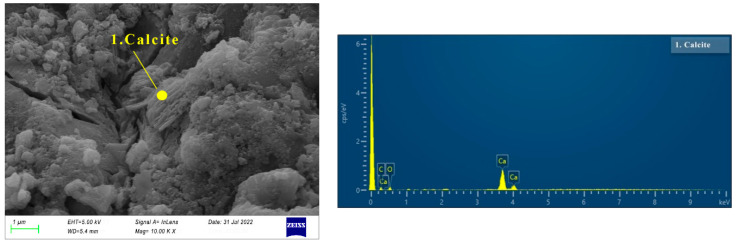
Micrographs of specimen N25 cured for 1 day.

**Figure 7:**
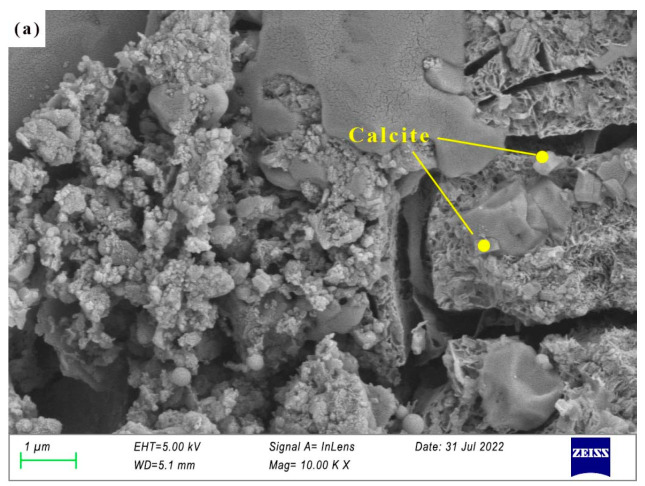

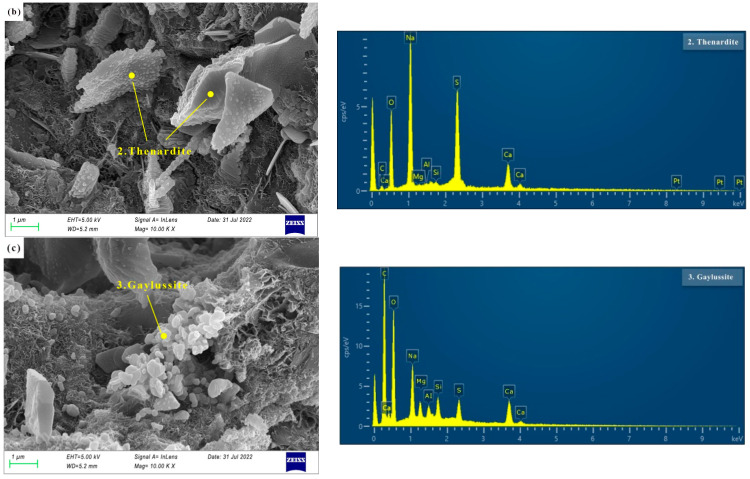
(**a**–**c**) Micrographs of specimen N25 cured for 3 days.

**Figure 8:**
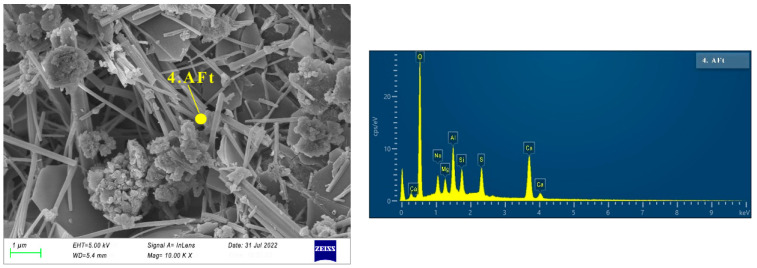
Micrographs of specimen N25 cured for 7 days.

**Figure 9:**
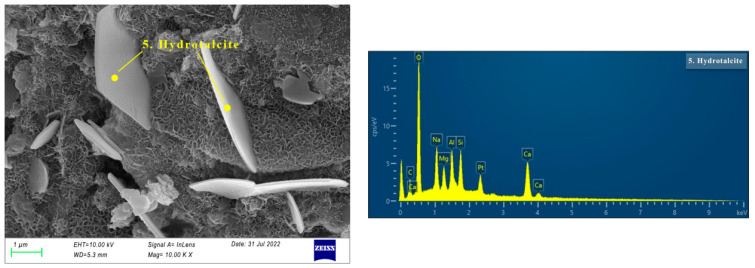
Micrographs of specimen N25 cured for 28 days.

The SEM images of specimen N25 are shown in Figure 6, Figure 7, Figure 8 and Figure 9. As can be seen from Figure 6, at the first day of hydration, many layered materials are intertwined with flocculated materials. The EDS shows that the substance is composed of Ca, C, O. Combined with the XRD analysis, the layered material is incomplete crystallized CaCO_3_, and the flocculated material was C-(A)-S-H gel. At 3 days of hydration, well-crystallized calcite appears (See Figure 7a). It can be seen from the EDS in Figure 7b that the substance is mainly composed of Na, S, O, which is thenardite that is not involved in the reaction. In addition, there are poorly crystallized gaylussites, as shown in Figure 7c. At 7 days of hydration (See Figure 8), the needle-like AFt, flocculent C-(A)-S-H gel, and lamellar hydrotalcite intersperse and overlap together. At 28 days of hydration (See Figure 9), the lamellar hexagonal crystalline hydrotalcite is interspersed in the honeycomb or reticular gel structure, forming a relatively dense hardened cement matrix. 

### 3.4. Production Cost, CO_2_ Emission, and Energy Consumption

**Figure 10:**
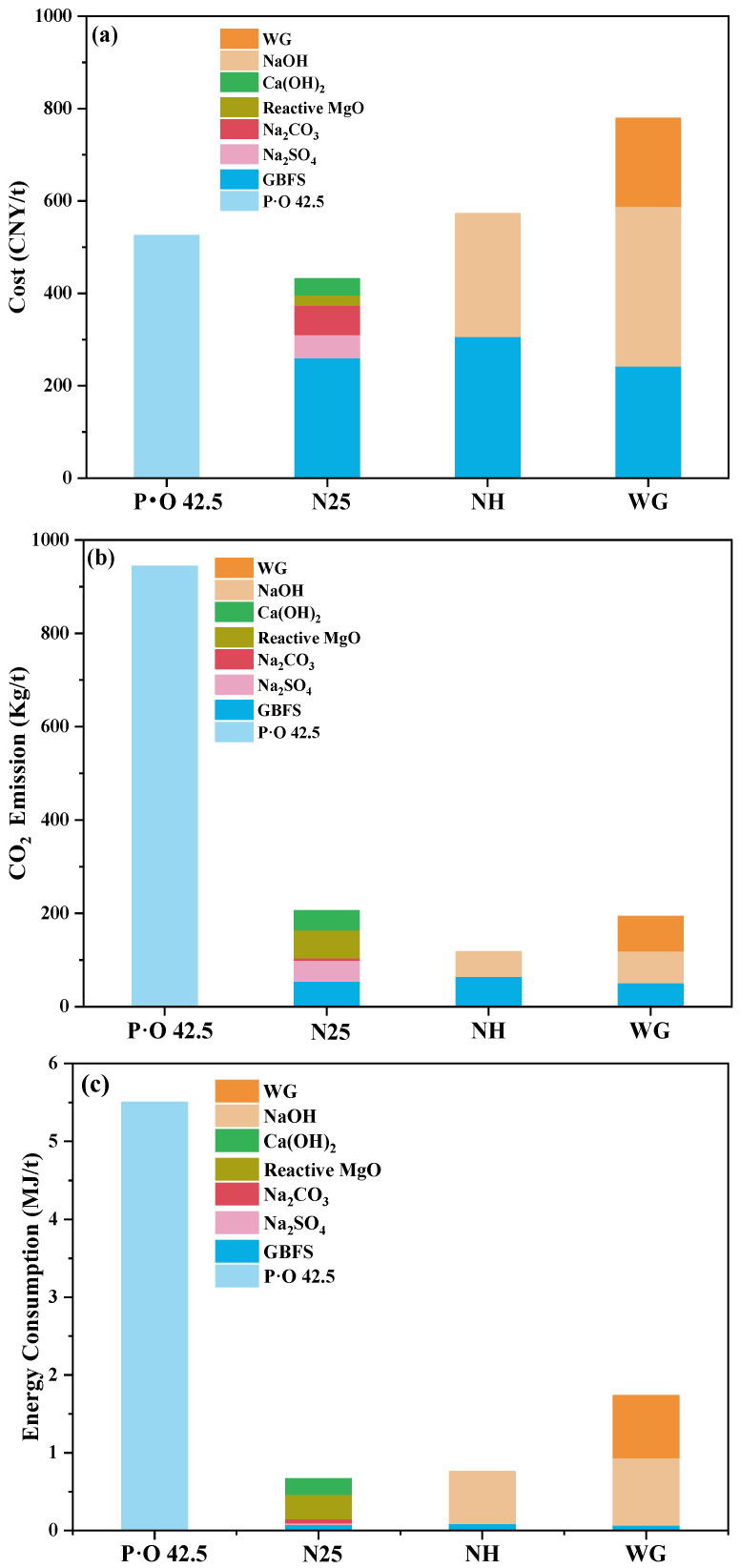
(**a**) Cost, (**b**) CO_2_ emission, (**c**) energy consumption of P·O 42.5, N25, NH, and WG.

P·O 42.5 of OPC and AAS cement activated by NH and WG with the same alkali equivalent of 5.4% were selected and the production cost, CO_2_ emission, and energy consumption were compared with specimen N25. The mortars were prepared according to the ratio of cementitious material:sand:water = 1:3:0.5. The compressive strength was measured to calculate the cost, CO_2_ emission, and energy consumption per unit strength. The production cost, CO_2_ emission, and the energy consumption of constituent raw materials of cementitious materials are shown in Table 9. The composition of the four cementitious materials is shown in Table 10. Based on Table 9 and Table 10, production cost, CO_2_ emission, and energy consumption for the preparation of 1 t cementitious materials are calculated, as shown in Figure 10. For simplifying the calculation, the transportation of raw materials, mixing, and hydration of mixtures were not quantified.

As shown in Figure 10a, the highest cost of preparing 1 t cementitious material is WG, while the lowest is N25. Compared with P·O 42.5, NH, and WG, the production cost of N25 is reduced by 12.7%, 20.0%, and 41.2%, respectively. As shown in Figure 10b, the highest CO_2_ emission of 1 t of cementitious material is P·O 42.5 and the lowest is NH. Compared with P·O 42.5, NH, and WG, the CO_2_ emission of N25 decreased by 77.6% and increased by 80.0% and 9.5%. As shown in Figure 10c, the highest energy consumption in the preparation of 1 t cementitious materials is P·O 42.5 and the lowest is N25. Compared with P·O 42.5, NH, and WG, the energy consumption of N25 decreased by 87.7%, 11.0%, and 61.1%.

The 28-day compressive strength of the mortar prepared by P·O 42.5 and the AAS cement by N25, NH, and WG, according to the same mix proportion, is shown in Table 11. Based on the amount of cementitious material used in preparing a group of mortar specimens, and the cost, CO_2_ emission, and energy consumption of 1t cementitious material, the cost, CO_2_ emission, and energy consumption of a group of mortar was calculated. These data are then divided by the strength value to get the cost, CO_2_ emission, and energy consumption per unit strength. This is shown in Table 11. It can be seen that the 28-day compressive strength of WG-activated AAS mortar is the highest, and NH-activated AAS mortar is the lowest. The highest cost per unit strength is NH and the lowest is N25. Compared with P·O 42.5, NH, and WG, the cost per unit strength of N25 is reduced by 14.9%, 31.9%, and 10.3%. The highest CO_2_ emission per unit strength is P·O 42.5 and the lowest is NH. Compared with P·O 42.5, NH, and WG, the CO_2_ emission per unit strength of N25 decreased by 78.1% and increased by 53.3% and 66.9%. The highest energy consumption per unit strength is P·O 42.5 and the lowest is N25. Compared with P·O 42.5, NH, and WG, the energy consumption of N25 is reduced by 88.0%, 24.1% and 40.7%.

Compared with NH and WG, the CO_2_ emission of N25 is higher. The main reason is that Ca(OH)_2_ and reactive MgO are used to improve the performance of the AAS cement activated by a weakly alkaline activator. The preparation of Ca(OH)_2_ and reactive MgO will emit a lot of CO_2_. In addition, the content of Ca(OH)_2_ and reactive MgO in the specimen of N25 are both at the highest value of 5%. Since the content of Ca(OH)_2_ and reactive MgO has little effect on the performance of AAS cement, except that a low content of Ca(OH)_2_ obviously prolongs the final setting time, adding a low content of Ca(OH)_2_ and reactive MgO can significantly reduce the CO_2_ emission. 

## 4. Conclusions

(1) Ca(OH)_2_ was the significant factor affecting the setting time of AAS cement activated by a weakly alkaline activator. Na_2_SO_4_ and Na_2_CO_3_ are significant factors affecting the flexural strength and compressive strength of AAS mortar, respectively. The interaction between Ca(OH)_2_ and Na_2_CO_3_ has a greater effect on the initial setting time than on the final setting time. That interaction has a greater effect on the 3-day compressive strength than that on the 28-day compressive strength.
(2) The reaction between the activators of Ca(OH)_2_ and Na_2_CO_3_ to form CaCO_3_ crystals is the main reason that affects the initial setting time and the early strength of AAS cement. The rapid reaction of Ca(OH)_2_ with Na_2_CO_3_ and Na_2_SO_4_ enhances the alkalinity of the AAS system and further promotes the hydration of GBFS. When an activator is composed of 7% Na_2_SO_4_, 4–5% Na_2_CO_3_, 3–5% Ca(OH)_2_, and 2–4% reactive MgO, the setting time of the AAS cement is reasonable and the mechanical property is good.
(3) There are more crystal phases in hydration products, namely calcite, AFt, and hydrotalcite. Na_2_SO_4_ and reactive MgO contribute to the formation of AFt and hydrotalcite, respectively. The effect of the crystal phases on dry shrinkage needs to be further studied.
(4) Compared with P·O 42.5 of OPC and AAS cement activated by NH and WG with the same alkali equivalent of 5.4%, the cost of producing 1 ton of cementitious material is reduced by 12.7%, 20.0%, and 41.2%, and energy consumption is reduced by 87.7%, 11.0%, and 61.1%. Compared with P·O 42.5, CO_2_ emission is reduced by 77.6%. The engineering application of AAS cement activated by weakly alkaline activators will bring good economic and environmental benefits.

## Figures and Tables

**Table 1:** Chemical composition of GBFS and reactive MgO (%).

Composition	GBFS	Reactive MgO
CaO	41.15	5.07
MgO	7.67	87.00
Al_2_O_3_	14.41	0.58
Fe_2_O_3_	0.49	0.99
SiO_2_	30.91	5.21
SO_3_	1.61	0.86
Na_2_O	0.75	0.02
K_2_O	0.57	0.06
Loss of ignition	2.44	0.21

**Table 2:** Factor level table.

Level	A	B	C	D	E	F
	Na_2_SO_4_ (%)	Na_2_CO_3_ (%)	Reactive MgO (%)	Ca(OH)_2_ (%)	Na_2_SO_4_ ×Ca(OH)_2_	Na_2_CO_3_ ×Ca(OH)_2_
1	3	2	2	3	1	1
2	5	3	3	4	2	2
3	7	4	4	5	3	3
4	9	5	5	6	4	4
5	7	4	3	4	5	5

**Table 3:** Orthogonal experimental design table.

Serial Number	Na_2_SO_4_ (%)	Na_2_CO_3_ (%)	Reactive MgO(%)	Ca(OH)_2_ (%)	Na_2_SO_4_ ×Ca(OH)_2_	Na_2_CO_3_ ×Ca(OH)_2_	n_Ca(OH)2_ /n_Na2CO3_	n_Ca(OH)2_ /n_Na2SO4_	n_Ca(OH)2_ /(n_Na2SO4_ + n_Na2CO3_)
N1	3	2	2	3	1	1	2.1	1.9	1
N2	3	3	3	4	2	2	1.9	2.6	1.1
N3	3	4	4	5	3	3	1.8	3.2	1.1
N4	3	5	5	6	4	4	1.7	3.8	1.2
N5	3	4	3	4	5	5	1.4	2.6	0.9
N6	5	2	3	5	4	5	3.6	1.9	1.2
N7	5	3	4	6	5	1	2.9	2.3	1.3
N8	5	4	5	4	1	2	1.4	1.5	0.7
N9	5	5	3	3	2	3	0.9	1.2	0.5
N10	5	4	2	4	3	4	1.4	1.5	0.7
N11	7	2	4	4	2	4	2.9	1.1	0.8
N12	7	3	5	3	3	5	1.4	0.8	0.5
N13	7	4	3	4	4	1	1.4	1.1	0.6
N14	7	5	2	5	5	2	1.4	1.4	0.7
N15	7	4	3	6	1	3	2.1	1.6	0.9
N16	9	2	5	4	5	3	2.9	0.9	0.7
N17	9	3	3	5	1	4	2.4	1.1	0.7
N18	9	4	2	6	2	5	2.1	1.3	0.8
N19	9	5	3	4	3	1	1.1	0.9	0.5
N20	9	4	4	3	4	2	1.1	0.6	0.4
N21	7	2	3	6	3	2	4.3	1.6	1.2
N22	7	3	2	4	4	3	1.9	1.1	0.7
N23	7	4	3	3	5	4	1.1	0.8	0.5
N24	7	5	4	4	1	5	1.1	1.1	0.6
N25	7	4	5	5	2	1	1.8	1.4	0.8

**Table 4:** Setting time of AAS paste and mechanical strength of AAS mortar.

Serial Number	Initial Setting Time (min)	Final Setting Time (min)	3-Day Flexural Strength (MPa)	3-Day Compressive Strength (MPa)	28-Day Flexural Strength (MPa)	28-Day Compressive Strength (MPa)
N1	80	140	5.1	21.9	6.2	26.9
N2	58	83	5.6	27.0	7.3	33.2
N3	49	77	5.9	26.9	7.6	34.7
N4	44	67	5.9	25.3	6.9	35.5
N5	71	105	6.1	27.4	7.7	35.0
N6	49	66	6.0	18.3	7.4	28.4
N7	47	53	6.7	25.8	7.6	36.8
N8	49	79	6.4	26.2	7.7	44.4
N9	59	77	6.6	31.5	7.8	36.7
N10	49	68	7.2	30.1	7.9	37.6
N11	51	73	6.2	19.4	7.3	29.6
N12	47	69	6.5	25.7	8.5	34.3
N13	51	88	7.2	27.8	8.0	39.0
N14	50	87	6.8	29.0	8.2	37.2
N15	43	66	6.7	30.1	7.6	35.6
N16	44	68	6.9	28.0	8.2	37.6
N17	45	76	7.7	27.5	8.5	34.8
N18	47	77	6.6	23.7	7.5	32.2
N19	81	151	7.3	29.8	7.7	36.2
N20	79	128	6.8	29.1	8.0	39.2
N21	52	72	6.2	18.0	8.2	31.5
N22	56	72	7.2	27.2	8.8	38.2
N23	49	99	7.0	28.0	8.9	41.1
N24	49	94	7.5	31.9	9.1	43.0
N25	47	64	7.9	30.6	9.0	44.6

**Table 6:** Interaction of B (Na_2_CO_3_) × D (Ca(OH)_2_) on initial setting time (min).

	D (Ca(OH)_2_)	D_1_	D_2_	D_3_	D_4_
B (Na_2_CO_3_)					
B_1_		80	48	49	52
B_2_		47	57	45	47
B_3_		64	55	48	45
B_4_		59	65	50	44

**Table 7:** Correction range value (R′) of mechanical strength of AAS mortar.

Factors	R′ of 3-Day Flexural Strength	R′ of 3-Day Compressive Strength	R′ of 28-Day Flexural Strength	R′ of 28-Day Compressive Strength
A (Na_2_SO_4_)	1.48	2.12	1.34	4.79
B (Na_2_CO_3_)	0.82	9.24	0.75	8.31
C (Reactive MgO)	0.15	0.86	0.37	5.36
D (Ca(OH)_2_)	0.51	3.20	0.64	3.37
A × D (Na_2_SO_4_ ×Ca(OH)_2_)	0.11	1.88	0.30	2.40
B × D (Na_2_CO_3_ ×Ca(OH)_2_)	0.43	2.99	0.30	2.25

**Table 8:** Results of specimens with good properties.

Serial Number	Initial Setting Time (min)	Final Setting Time (min)	3-Day Flexural Strength (MPa)	3-Day Compressive Strength (Mpa)	28-Day Flexural Strength (Mpa)	28-Day Compressive Strength (Mpa)
N13	51	88	7.2	27.8	8.0	39.0
N23	49	99	7.0	28.0	8.9	41.1
N24	49	94	7.5	31.9	9.1	43.0
N25	47	64	7.9	30.6	9.0	44.6
N8	49	79	6.4	26.2	7.7	44.4

**Table 9:** Production cost, CO_2_ emission, and energy consumption of constituent raw materials.

	Cost (CNY/t) ^c^	CO_2_ Emission (kg/t) ^d^	Energy Consumption (GJ/t) ^d^	Reference
Na_2_CO_3_	1580	111	1.3	[36]
Na_2_SO_4_	720	640	0.3	[37,38]
NH	3800	750	9.5	[39]
Ca(OH)_2_	700	830	4	[36]
Reactive MgO ^a^	450	1200	6.3	
P·O 42.5	525	944	5.5	[17,40]
GBFS	330	70	0.1	[39]
WG ^b^	1100	425	4.6	[41]

^a^ This information was provided by the manufacturer. ^b^ The modulus of WG was 3.2. ^c^ Cost data obtained from Chinese market. ^d^ CO_2_ emission and energy consumption data obtained from [17,36,37,38,39,40,41].

**Table 10:** The composition of P·O 42.5 and AAS cement of N25, NH, WG (%).

Cementitious Material	GBFS	Na_2_SO_4_	Na_2_CO_3_	Ca(OH)_2_	Reactive MgO	P·O 42.5	NH	WG
P·O 42.5						100		
N25	79	7	4	5	5			
NH-AAS cement	93						7	
WG AAS cement ^a^	73.6						9.1	17.3

^a^ The modulus of raw WG was 3.2, adjusted to 1.2 by adding NH.

**Table 11:** Compressive strength and the cost, CO_2_ emission, and energy consumption per unit strength.

Cementitious Material	28-Day Compressive Strength (MPa)	Cost/Strength (CNY/(MPa))	CO_2_ Emission/Strength (kg/(MPa))	Energy Consumption/Strength (GJ/(MPa))
P·O 42.5	43.5	0.0054	9.77	0.057
N25-AAS cement	44.6	0.0046	2.14	0.007
NH-AAS cement	38.0	0.0068	1.39	0.009
WG	68.0	0.0052	1.28	0.011

## Data Availability

Not applicable.
